# Cost-effectiveness and cost-utility of an Acceptance and Commitment Therapy intervention vs. a Cognitive Behavioral Therapy intervention for older adults with anxiety symptoms: A randomized controlled trial

**DOI:** 10.1371/journal.pone.0262220

**Published:** 2022-01-26

**Authors:** Maartje Witlox, Vivian Kraaij, Nadia Garnefski, Ernst Bohlmeijer, Filip Smit, Philip Spinhoven

**Affiliations:** 1 Faculty of Social and Behavioural Sciences, Section of Clinical Psychology, Institute of Psychology, Leiden University, Leiden, The Netherlands; 2 Department of Psychology, Health and Technology, University of Twente, Enschede, The Netherlands; 3 Department of Mental Health & Prevention, Netherlands Institute of Mental Health and Addiction, Utrecht, The Netherlands; Brown University, UNITED STATES

## Abstract

**Background:**

A previous randomized controlled trial in older adults with anxiety symptoms found no differences between a brief blended Acceptance and Commitment Therapy (ACT) intervention and brief face-to-face Cognitive Behavior Therapy (CBT) regarding anxiety symptom severity at posttreatment and 12-month follow-up. A health-economic evaluation comparing these interventions has not yet been conducted.

**Objective:**

This study examined the one-year cost-effectiveness and cost-utility of blended ACT compared to face-to-face CBT for older adults with anxiety symptoms.

**Methods:**

The economic evaluation was embedded in a randomized controlled trial comparing blended ACT to CBT in 314 older adults with mild to moderately severe anxiety symptoms. Data were collected at baseline and 3, 6 and 12 months post baseline. For the cost-effectiveness analysis, treatment response was defined as a reliable improvement in anxiety symptom severity (measured with the Generalized Anxiety Disorder-7) between baseline and 12-month follow-up. To assess cost-utility, quality-adjusted life years (QALYs) were computed using EuroQol-5 Dimensions-5 Levels-5 utility scores. Analyses took the societal perspective, including both healthcare costs and productivity costs. Incremental cost-effectiveness ratios were calculated using 2500 bootstraps of seemingly unrelated regression equations of costs and effects. Sensitivity analyses were performed to assess the robustness of the findings.

**Results:**

Differences between the blended ACT group and CBT group in treatment response and QALYs were statistically insignificant and clinically irrelevant. The ACT intervention was associated with an average per-participant cost reduction of €466 ($593) compared to CBT, which resulted from lower productivity costs in the blended ACT group. From a healthcare perspective, the ACT intervention was associated with higher costs (by €71 ($90)) than CBT.

**Conclusions:**

The results do not indicate that from a health-economic perspective blended ACT should be preferred over CBT in the treatment of older adults with anxiety symptoms. The findings support a model of shared decision making, where clinicians and patients collaboratively decide on the preferred intervention, based on ethical-medical, practical and personal considerations.

**Trial registration:**

Netherlands Trial Register: TRIAL NL6131 (NTR6270); https://www.trialregister.nl/trial/6131.

## Introduction

Anxiety symptoms are the most prevalent mental health problem in older adults (55 years and over) and have an adverse impact on subjective well-being, quality of life, physical health and everyday functioning [1–5]. In addition, anxiety symptoms are associated with increased costs stemming from healthcare utilization and productivity losses [6, 7]. Reducing the personal and societal burden of anxiety in later life should therefore be a public health priority, especially in light of the unprecedented growth of the proportion of older adults worldwide that will confront mental health care institutions with an increasing number of older patients [8]. To advance the evidence-based treatment of anxiety in later life, psychological interventions should be rigorously evaluated in older study samples.

So far, most trials in anxious older adults have focused on face-to-face cognitive behavioral therapy (CBT) [9] and multiple clinical guidelines refer to CBT as the preferred treatment option for older adults with anxiety symptoms [10–12]. Recently, studies have indicated that online and blended CBT interventions are also effective at reducing anxiety symptom severity in older adults, which is promising as scalable internet-based interventions are likely to become crucial in providing this large patient population with adequate psychological treatment [13–16]. Although clinical trials so far confirm the effectiveness of CBT interventions for anxiety in later life, it is important to also examine other treatment approaches for anxious older adults. First, when compared to active control conditions, effect sizes favoring CBT are small in samples of older adults with anxiety symptoms and/or disorders [17]. Furthermore, some evidence suggests that CBT is less effective in older adults than in younger samples [17, 18].

A promising treatment alternative is Acceptance and Commitment Therapy (ACT), a so called third-wave cognitive behavioral therapy. ACT is a transdiagnostic treatment that focuses on increasing acceptance-based emotion regulation and the identification and prioritization of intrinsic values and related behavior change [19]. The main goal of ACT is not to merely reduce psychological symptoms, but rather to stimulate people to start living a more meaningful, fulfilling life. ACT might be especially suitable for older patient populations because it aligns with age-related tendencies to be more accepting towards (negative) emotions and reevaluate personal values [20, 21].

The present study evaluates the cost-effectiveness and cost-utility of a brief blended ACT intervention (a combination of an online self-help module with face-to-face sessions with a mental health counselor) compared to brief face-to-face CBT for older adults with anxiety symptoms. The cost-effectiveness analysis (CEA) presents effects in terms of treatment response (i.e., long-term anxiety symptom improvement) and the cost-utility analysis (CUA) in terms of quality adjusted life years (QALYs). This health economic evaluation was embedded in a randomized controlled trial (RCT) that found no difference between these two interventions regarding anxiety symptom improvement at posttreatment and 12-month follow-up. On a within-group level, participants in both conditions showed significant reductions in anxiety symptom severity from baseline to posttreatment that were sustained at the 6- and 12-month follow-ups [22]. This RCT was the first large-scale study to evaluate an ACT intervention for older adults with anxiety symptoms. The results are promising and suggest that blended ACT is at par with CBT.

The cost-effectiveness analysis (CEA) and cost-utility analysis (CUA) in the current study can add valuable insights into the comparative effects of blended ACT and CBT for older adults with anxiety symptoms. First, as the cost-utility analysis considers treatment effects in terms of quality of life, this study provides insight into the broader, transdiagnostic effects of the interventions. Second, the analyses will shed light on how the two interventions affect healthcare utilization and work productivity. Lastly, the integration of data on treatment effects and associated costs may inform policy making as it could indicate if the ACT intervention is likely to achieve its effects at similar or lower societal costs than CBT, which is currently the gold standard treatment for anxiety in later life [9–12]. This study will be the first health-economic evaluation of an ACT intervention for older adults. Furthermore, to the best of our knowledge, it will also be the first such evaluation of ACT compared to CBT in any patient population.

## Methods

### Research design

The health-economic evaluation was embedded in a study into the effectiveness of a brief blended ACT intervention compared to brief face-to-face CBT for older adults with anxiety symptoms. This study was a pragmatic cluster-randomized, controlled, single-blind trial, comparing the relative merits of both interventions over a period of 12 months.

Randomization took place at the level of mental health counselors working at general practitioner’s (also sometimes called primary care physician) offices. This created clusters of participants that all received the same treatment from the same counselor.

Assuming a mean cluster size of five participants per mental health counselor at posttreatment, an intraclass correlation of 0.01 and a coefficient of variation of 0.30, 18 mental health counselors (or 90 participants) were required in each of the two study arms to detect a between-group difference on the Generalized Anxiety Disorder-7 (GAD-7) at posttreatment with a medium effect size (Cohen *d* = 0.45), a 2-tailed α of .05, and a power of 0.80 [23]. Anticipating a dropout rate of 25%, we aimed to include 240 participants at baseline.

Participating mental health counselors were randomized to either blended ACT or face-to-face CBT using a block-randomization table (blocks of four) that was created by an independent researcher. This table was concealed from the other researchers. The randomization table was created by randomizing the six different possible sequences of two conditions in blocks of four. Each time four new mental health counselors had registered for participation, the independent researcher informed the main researcher about the randomization allocation of these four counselors (N.B., the main researcher received the allocation status of each block of four counselors at the same time. If randomization status would have been disclosed separately for each new counselor that registered for participation, the main researcher would have been able to predict the status of each third and or/fourth counselor within a block). Consequently, the main researcher contacted the counselors to inform them about their allocation.

The main researcher, mental health counselors, and participants were not blind for treatment allocation. However, participants were not informed about whether the intervention they were provided with was the experimental condition (blended ACT) or active control condition (CBT). The study was registered in the Netherlands Trial Register (NL6131) and approved by the medical ethics committee of Leiden University Medical Center (LUMC; P16.248). The study protocol that describes the methods in detail has been published elsewhere [23].

### Participants and procedure

From November 2017 to March 2019 participants were recruited in 38 general practices located in the Netherlands (the last 12-month follow-up assessment was completed in March 2020). The practices employed (one or more) mental health counselor(s) that provided treatment to the study participants. Patients aged 55–75 years from the participating general practices were sent an information and invitation letter and could register for participation on a study website, after which they entered a screening procedure consisting of both self-report online questionnaires and a telephone interview conducted by trained research assistants. The following inclusion criteria were used: age 55–75 years, presence of mild to moderately severe anxiety symptoms as measured with the Generalized Anxiety Disorder-7 (GAD-7; scores between 5 and 15 [24]); mastery of the Dutch language, internet access and motivation to spend 2.5 hours per week on the intervention. Exclusion criteria were: severe cognitive impairment or unstable severe medical condition(s); very mild or severe anxiety symptoms ((GAD-7) score < 5 / > 15 [24]); severe depressive symptomatology (Patient Health Questionnaire-9 (PHQ-9) score ≥ 20 [25]); psychological or psychopharmacological treatment within the last three months, with the exception of stable benzodiazepine or SSRI use (assessed during the telephone interview); severe functional impairment (score ≥ 8 on two or three Sheehan Disability Scale (SDS) domains [26]; assessed during the telephone interview); high suicide risk (M.I.N.I.-Plus [27]); substance use disorder (M.I.N.I.-Plus; assessed during the telephone interview); lifetime diagnosis of bipolar disorder or schizophrenia (medical record or M.I.N.I.-Plus (conducted during telephone interview).

Eligible participants were informed about their treatment allocation by the main researcher after they had given online informed consent and completed the baseline assessment. Participants completed 4 main assessments: baseline (T0), posttreatment (T1; 3 months after baseline), 6 months after baseline (T2) and 12 months after baseline (T3). These assessments consisted predominantly of online self-report questionnaires. T0, T1 and T3 additionally included a telephone interview, conducted by trained and supervised research assistants that were blind to randomization status of the participants.

### Interventions

#### Blended Acceptance and Commitment Therapy

Participants in the blended ACT condition received a combination of four face-to-face sessions with the mental health counselor at their general practice and internet self-help in the form of the online module “Living to the Full” [28], which was proven to be effective in reducing psychological distress in adults [29, 30]. The module is comprised of nine lessons that revolve around the six core processes of ACT: acceptance, cognitive defusion, contact with the present moment, self-as-context, values and committed action. Completing the lessons in time required the participants to spend 15 to 30 minutes on the module each day. During the face-to-face sessions (which lasted 30 to 40 minutes), the mental health counselors followed a treatment protocol that was developed by the authors of “Living to the Full”. The intervention was delivered in a period of 9 to 12 weeks (e.g., the allowed period between the first and last face-to-face session was 9 to 12 weeks).

#### Brief face-to-face cognitive behavioral therapy

Participants received protocolized CBT, consisting of four face-to-face sessions (30 to 40 minutes; period between first and last session ranged between 9 and 12 weeks) and homework exercises that required between 15 and 30 minutes on a daily basis. The protocol contained 12 different worksheets that mainly focused on identifying thinking errors and reducing anxiety-related avoidance behavior. Most of the worksheets were focused on specific types of anxiety (panic, worrying, social anxiety). Some focused on common side effects of anxiety (sleeping problems, physical tension). After the intake session, the counselor and participant set treatment goals and homework exercises were planned and prepared. In the second and third session, homework exercises were evaluated and key exercises/information repeated. The last session was dedicated to evaluating progress and formulating a relapse prevention plan.

#### Therapists

Treatment was provided by 40 mental health counselors working at general practices, who were randomized to either provide participants with blended ACT (n = 20) or with CBT (n = 20). Since approximately 2008, general practices in the Netherlands have employed mental health counselors that provide treatment to patients with mild to moderately severe psychological problems, preventing these patients from being referred to (specialized) mental health care institutions, which often have long waiting lists [31]. This position is fulfilled by mental health professionals from diverse educational and professional backgrounds. In the current study, most counselors were psychologists (n = 13), social psychiatric nurses (n = 14) or social workers (n = 5). Mental health counselors received a six hour in-person training in working with the treatment protocol for the treatment they were allocated.

### Outcome measures

#### Cost-effectiveness: Treatment response

Health benefit in the CEA was measured in terms of anxiety symptom improvement over 12 months.

Anxiety symptom severity was measured with the GAD-7, a widely used seven-item anxiety screener with well-established psychometric qualities (total scores 0–21, higher scores indicating greater symptom severity) [24]. For the CEA, long-term treatment response was operationalized as reliable improvement of anxiety symptom severity between baseline and the 12-month follow-up. For each participant, a so-called reliable change index (RCI) was computed by dividing the difference between GAD-7 scores at baseline and 12-month follow-up by the standard error of difference (the error variance in a set of scores resulting from the unreliability of the used scale) [32]. RCIs lower than -1.96 indicate reliable symptom improvement [33]. Using the RCIs, we created the final binary treatment response variable (0 = no treatment response (i.e., RCI > -1.96); 1 = treatment response (i.e., RCI < -1.96)).

#### Cost-utility: Quality of life

For the CUA, QALYs were computed from participants’ responses on the 5-level EQ-5D (EQ-5D-5L) questionnaire [34] at baseline and 3-, 6- and 12-month follow-up. The EQ-5D-5L assesses self-reported quality of life at the day of assessment using five domains (mobility, self-care, usual activities, pain/discomfort, and anxiety/depression). Severity of problems in each domain can be scored from 1 to 5. A total of 3125 unique health states can be defined, by combining the responses for the five domains into a 5-digit number (ranging from ‘11111’ meaning no problems at all to ‘55555’ meaning extreme problems in all five dimensions) [34]. These 5-digit numbers can be translated into preference-based utility scores (using the Dutch social tariff [35]), anchored between 0 (health state equivalent to being dead) and 1 (full health). The utility scores at the four measurement points were then used to calculate QALY gains using the area under the curve method, which assumes that change in the utility scores occurs linearly in the periods between the assessments. This method weighs the 12-month study period according to the utility scores at each measurement point.

#### Costs

For each participant, healthcare costs and costs stemming from productivity losses over the preceding four weeks were collected at baseline and 6- and 12-month follow-up with the Trimbos Institute and Institute of Medical Technology Assessment Questionnaire for Costs Associated with Psychiatric Illness (TiC-P) [36]. Table 1 in S1 Appendix lists all the assessed health care services. For each service, participants indicated if they had used it during the preceding four weeks, and if so, how many times they used it. They were also asked how many days they had used prescribed medication for depression, anxiety, pain and sleeping problems. To assess productivity losses, both absenteeism (“How many days did you not work due to health problems”) and presenteeism (“How many days did you work while not feeling well?) were assessed in relation to paid work, voluntary work and informal care. The TiC-P is the most widely used health service receipt interview in the Netherlands and its reliability in assessing information on health care utilization and productivity losses in patients with mild to moderate mental health conditions has been established [37]. Cumulative costs over the total 12-month study period were calculated using interpolation, assuming a linear trend in costs during the periods between the measurement points.

#### Direct medical costs (healthcare utilization)

Costs associated with healthcare utilization were computed by multiplying health service units (e.g., visits, consults) with their standard unit cost price [38] according to the Dutch manual for economic evaluations in healthcare (see S1 Appendix). Standard unit cost prices reported in this manual were calculated using various sources, including bottom-up micro costing studies, and top-down studies using information from national databases [38]. Medication costs were calculated as the average cost price per standard daily dose (using prices of the 5 most frequently prescribed medications in each of the four categories), as reported in the Dutch Pharmaceutical Compass [39], multiplied by the number of prescription days, plus pharmacists’ dispensing costs per monthly prescription.

#### Direct non-medical costs (travel costs)

Travel costs incurred in the context of visiting health services were calculated as the average distance of a return-trip to and from a health service (according to the Dutch manual for economic evaluations in healthcare[38]) multiplied by the costs per kilometer (€0.19; as stated in the same manual [38]) (See S2 Appendix).

#### Indirect costs (productivity losses)

Productivity losses at paid work, voluntary work and informal care due to absenteeism and presenteeism were assessed. Productivity loss due to presenteeism was computed by multiplying the number of workhours for which the participant reported reduced productivity with a fraction reflecting the reported level of inefficiency during those hours. Total costs due to productivity losses were calculated by multiplying the amount of work hours lost by the standard economic cost prices for paid work (€37.11) and unpaid work (€14.95) as reported in the Dutch manual for economic evaluations in healthcare [38] (see S1 Appendix).

#### Intervention costs

Participants in both conditions had four sessions with the mental health counselor (total costs €73). The additional per-participant costs of the online ACT module were €49, based on the market price of the module as determined by the current provider.

### Statistical analysis

Analyses were conducted using R statistical Software [40] and Stata, version 13.1 [41].

#### Imputation

All analyses followed the intention-to-treat principle, which required imputing missing values. Missing data were imputed using multiply imputed chained equations (MICE), with the predictive mean matching procedure, where the missing outcome for a non-respondent (so called ‘*recipient’*) is imputed with the observed outcome from a respondent (‘*donor’*) with a comparable predicted mean outcome [42]. This procedure ensures that the imputed data have plausible values. In the current study, this meant that imputed costs were not negative, and imputed utility scores fell between 0 and 1. We included the following baseline variables into the imputation, because they were predictive of missingness and/or associated with the outcome variable(s): sex, education level, age, depression symptom severity, presence of DSM-V anxiety disorder.

#### Cost-effectiveness and cost-utility analyses

To examine between-group differences in treatment response, we calculated a risk difference using a linear probability model that accounted for the clustered structure of the data (i.e., clusters of participants receiving treatment from the same therapist). Cumulative QALY gains and costs in both conditions were compared using linear regression, also accounting for the clustering in the data. We do not report a p-value for the between-group costs differences, because cost data usually have a high variance and therefore require very large sample sizes to detect a statistically significant difference, for which this trial was not powered [43]. Costs in euros were converted to US dollars using purchasing power parities (PPP) as reported by the Organisation for Economic Co-operation and Development (OECD) for the reference year 2019 [44]. PPPs take into account both the currency exchange rate and the differences in buying power between the two countries in that year. The CEA and CUA were conducted from the societal perspective, which means that both medical costs and costs stemming from productivity losses were included in the cost calculations. In both analyses, incremental cost-effectiveness ratios (ICERs) were calculated as the between-group cost difference divided by the between-group effect difference. The ICER reflects the additional costs associated with blended ACT per additional unit of effect gained. Cost- and effect differences between the conditions were obtained simultaneously from seemingly unrelated regression equations (which allows the residuals of the two equations to be correlated, thereby producing more efficient estimates). To capture the stochastic uncertainty in the ICERs due to sample error, the seemingly unrelated regression equations models were bootstrapped 2500 times and the mean ICER of each bootstrap step was plotted on a cost-effectiveness plane. This produces estimates of the probability that 1) compared to CBT, blended ACT results in better health for more costs (northeast quadrant); 2) blended ACT is dominated by CBT because it is associated with less health gains and higher costs (northwest quadrant); 3) compared to CBT, blended ACT produces less health gains for lower costs (southwest quadrant); 4) blended ACT is the dominant intervention, because compared to CBT better outcomes for lower costs are obtained (southeast quadrant). Besides the arrhythmic means of the bootstrapped cost-differences and effect-differences, the median cost- and effect differences were also calculated to better reflect that the underlying cost and effect data may not be normally distributed.

Acceptability curves were created to visualize the probability that blended ACT is cost-effective compared to CBT, for a range of willingness-to-pay (WTP) threshold per gained health unit. As there are no established willingness-to-pay ceilings available for the outcome in the CEA, curves were only created for the CUA. Research in the Netherlands has showed that people are willing to pay €53,000/QALY for another person, which rises to €83,000/QALY if it concerns themselves or a relative. Therefore, we used threshold values of €50,000 and €80,000 per QALY [45]. To test the robustness of the results, we conducted three sensitivity analyses. First, we repeated the analyses on a dataset imputed with the expectation maximization (EM) method, to assess the influence of imputation method on the results. Second, we conducted per-protocol analyses, in which we only included participants that attended either 3 or 4 face-to-face sessions (ACT n = 100, CBT n = 126). Lastly, we performed a cost-effectiveness and cost-utility analysis from a healthcare perspective, which only included healthcare costs.

## Results

**Fig 1:**
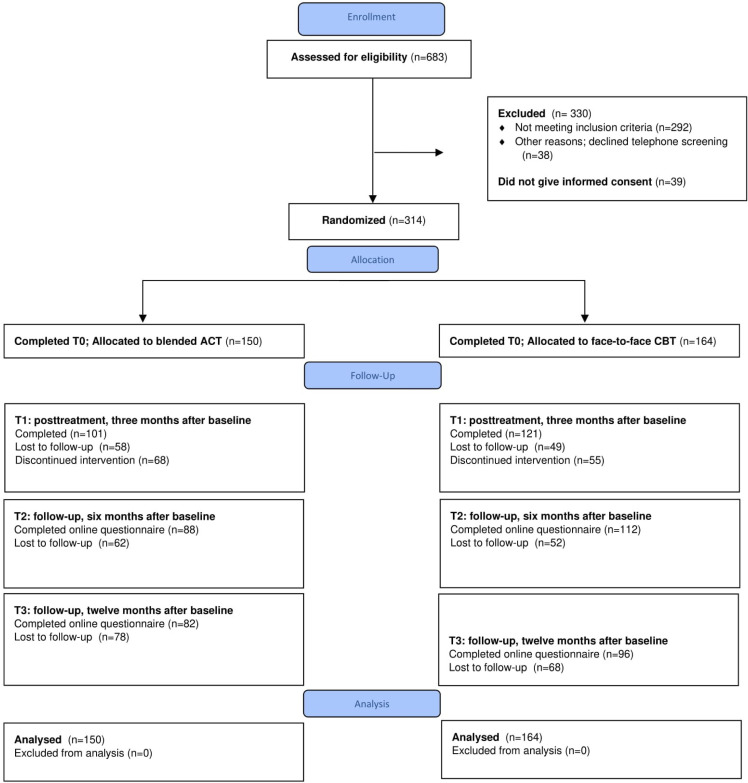
Flowchart of study participants.

A total of 40 mental health counselors participated in the study and were randomized to provide participants with either blended ACT (n = 20) or face-to-face CBT (n = 20). Mean cluster size (i.e., average number of participants treated by the same mental health counselor) at baseline was 7.85 (SD = 4.28, range 0–18). At posttreatment, mean cluster size was 5.55 (SD = 2.91 range 0–11). See Fig 1 for the participant flow diagram. A total of 314 participants gave informed consent and completed the baseline assessment: 150 were allocated to blended ACT condition, 164 to CBT. The difference in sample size between the two conditions stems from the cluster-randomized design; fourteen more participants were recruited from the general practices that employed mental health counselors who were randomized to the face-to-face CBT condition. Table 1 presents baseline demographic and clinical characteristics for the participants in the two conditions and the total study sample. We did not observe any clinically relevant differences between the conditions at baseline, suggesting that there was no baseline imbalance between the conditions. At first follow-up, T1, 71% (n = 222) of the participants completed the assessment (ACT 67%, CBT 74%); at T2, 64% (n = 200; ACT 59%, CBT 68%), and at T3, 57% (n = 178; ACT 55%, CBT 59%). The proportion of participants who did not complete one or more of the follow-up assessments did not differ between the conditions (*χ*^*2*^ (1) = 1.2, *P* = .27). Loss to follow-up was associated with gender: 55.74% of male participants did not complete one or more assessments, compared to 44.27% of female participants (*χ*^*2*^(1) = 3.9, *P* = .048). No adverse events were reported by any of the participants.

**Table 1:** Baseline characteristics of study sample.

Characteristics	Blended ACT (*n* = 150)	CBT (*n* = 164)	Total sample (*n* = 314)
Age (years), M (SD), [range]	62.75 (5.69)	63.33 (5.71)	63.06 (5.70)
	[55–75]	[55–75]	[55–75]
Sex, n (%)			
Female	100 (66.67)	92 (56.08)	192 (61.15)
Male	50 (33.33)	72 (43.92)	122 (38.85)
Nationality, n (%)			
Dutch	149 (99.33)	159 (96.96)	308 (98.01)
Dutch and other	0 (0.00)	5 (3.04)	5 (1.59)
Other	1 (0.77)	0 (0.00)	1 (0.40)
Education^a^, n (%)			
Low	22 (14.67)	15 (9.15)	37 (11.78)
Middle	70 (44.67)	74 (45.12)	144 (45.86)
High	56 (37.33)	74 (45.12)	130 (41.40)
Unknown	2 (0.63)	1 (0.61)	3 (0.96)
Relational status, n (%)			
Married/in a romantic relationship	120 (80.00)	129 (78.66)	249 (79.30)
Not married/in a romantic relationship	30 (20.00)	35 (21.34)	65 (20.70)
Work status, n (%)			
Paid employment	77 (51.33)	76 (46.34)	153 (48.73)
Voluntary work	49 (32.67)	56 (34.15)	105 (33.44)
No work	53 (35.33)	59 (35.98)	112 (35.67)
Living situation, n (%)			
Alone	36 (24.00)	39 (23.78)	75 (23.89)
With partner	97 (64.67)	103 (62.80)	200 (63.69)
With children	11 (7.33)	13 (7.93)	24 (7.64)
With partner and	6 (4.00)	8 (4.88)	14 (4.46)
children			
Other	0 (0.00)	1 (0.61)	1 (0.32)
Community dwelling	150 (100)	164 (100)	314 (100)
Somatic comorbidity, n (%)			
No somatic problems	29 (19.33)	32 (19.51)	61 (19.43)
One or more somatic	121 (80.67)	132 (80.49)	253 (80.57)
problems			
Medication use, n (%)			
Antidepressants	15 (9.15)	12 (8.00)	27 (8.60)
Anxiolytics	12 (7.32)	19 (12,67)	31 (9.87)
Sleeping medication	23 (14.02)	17 (11.33)	40 (12.74)
Pain medication	21 (12.80)	17 (11.33)	38 (12.10)
Anxiety disorder^b^, n (%)			
Any anxiety disorder	42 (28.00)	39 (23.78)	81 (25.80)
No anxiety disorder	108 (72.00)	125 (76.22)	233 (74.20)

Note.

^a^ High education level includes completed higher vocational education or university education. Middle education level includes a completed secondary school or intermediate vocational education. Low education level includes completion of primary school and/or secondary school.

^b^ Anxiety disorder diagnoses were established with the MINI-PLUS during a telephone diagnostic interview conducted by trained research assistants.

### Effects and costs

In S4 Appendix, reported units of healthcare utilization and reported days of absenteeism and presenteeism are presented. Table 2 contains the mean healthcare-, productivity- and total societal costs and mean anxiety symptom severity and utility values at the different measurement points for both treatment conditions.

**Table 2:** Mean costs and outcomes by condition over assessments.

Costs and outcomes	Baseline		Posttreatment		Follow-up		Follow-up	
			(3 months)		(6 months)		(12 months)	
	ACT	CBT	ACT	CBT	ACT	CBT	ACT	CBT
Costs, mean (SD)								
Healthcare costs	€100 (186)	€88 (123)	-	-	€92 (169)	€106 (136)	€108 (225)	€87 (129)
Productivity costs	€106 (256)	€179 (483)	-	-	€69 (197)	€125 (503)	€134 (470)	€130 719)
Total societal costs	€206 (329)	€267 (502)	-	-	€161(277)	€231 (526)	€242 (520)	€218 765)
Outcomes, mean (SD)								
Anxiety symptom	8.2 (4.1)	8.8 (4.2)	4.3 (3.7)	4.5 (3.5)	4.8 (3.2)	5.7 (3.6)	4.5 (4.0)	4.8 (3.6)
severity								
Utility value	.75 (.15)	.75 (.16)	.79 (.20)	.78 (.20)	.82 (.14)	.81 (.15)	.82 (16)	.81 (15)

*Note*. Costs were measured with the Trimbos Institute and Institute of Medical Technology Assessment Questionnaire for Costs Associated with Psychiatric Illness (TiC-P), on which participants reported their healthcare utilization and work productivity losses during the four weeks prior to the assessment. Anxiety symptom severity was measured with the Generalized Anxiety Disorder-7, a self-report measure that assesses anxiety symptoms during the preceding two weeks (range 0–21). Utility values were assessed with the EQ-5D-5L, which measures self-reported quality of life at the day of assessment.

#### Treatment response

In the blended ACT group 54 out of 150 (36%) participants were considered treatment responders, as they showed reliable improvement of anxiety symptoms between baseline and the 12-month follow-up. In the CBT group this was the case for 70 out of 164 (43%) participants. The between-group risk difference (i.e., the incremental effect) was 0.36–0.43 = -0.07 [95% CI: -0.17 to 0.04], which was not statistically significant (SE = 0.06, z = 1.13, *P* = .26).

#### Quality of life

Average quality of life utility values in the blended ACT group were .75 at baseline, .79 at 3-month follow-up, .81 6-month follow-up and .82 at 12-month follow-up. In the CBT group average scores were .75 at baseline, .78 at 3-month follow-up, .81 at 6-month follow-up and .81 at 12-month follow-up. This shows that health-related quality of life increased over time in both conditions. Cumulative QALYs were 0.797 in the CBT-group and 0.804 in the ACT-group. The 0.007 [95% CI: -0.22 to 0.04] between-group difference in cumulative QALYs was statistically nonsignificant (SE = .02, t = -0.46, *P* = .65) and fell below the established threshold for the minimal clinically relevant difference for the EQ-5D of 0.074 [46].

#### Healthcare costs

In the blended ACT group, the average per-participant healthcare costs were €100 at baseline, €92 at 6-moth follow-up and €108 at 12-month follow-up. The average healthcare costs per participant in the CBT group were €88 at baseline, €105 at 6-month follow-up and €87 at 12-month follow-up. Cumulative healthcare costs as incurred over the 12-month follow-up (including intervention costs of €73 for CBT and €112 for ACT) were €1300 in the ACT group and €1233 in the CBT group. The between-group difference (i.e., incremental costs) in total cumulative costs was €67 [95% CI: -€278 to €412] ($85).

#### Costs stemming from productivity losses

In the blended ACT group, average costs stemming from productivity losses were €106 at baseline, €69 at 6-month follow-up and €134 at 12-month follow-up. For the CBT group average costs were €179 at baseline, €125 at 6-month follow-up and €130 at 12-month follow-up. Cumulative costs per participant between baseline and 12-month follow-up were €1133 in the blended ACT group and €1681 in the CBT group. Cumulative costs over the study period were €548 [95% CI: -€1160 to €64] (-$698) lower in the ACT condition.

#### Total costs from the societal perspective

The average cumulative costs per participant from a societal perspective were €2433 in the blended ACT group and €2914 in the CBT group. Cumulative societal costs were thus lower in the ACT group, by €480 [95% CI: -€1190 to €229] (-$611).

### Cost-effectiveness and cost-utility

Table 3 summarizes the results of the main cost-effectiveness and cost-utility analyses and the sensitivity analyses: the mean incremental costs and effects from the 2500 bootstraps and the distribution of the bootstrapped ICERs over the quadrants of the cost-effectiveness plane Additionally, in S4 Appendix we present the median of the bootstrapped incremental costs and effects. The cost-effectiveness planes and acceptability curves of the sensitivity analyses are presented in S5 Appendix.

**Table 3:** Result of the main analyses (cost-effectiveness and cost-utility) and sensitivity analyses.

				Distribution of ICERs over the quadrants, %			
Analysis	Incr. Cost (ACT-CBT)	Incr. Effect (ACT-CBT)	ICER	NE	NW	SW	SE^2^
Base case CEA	-€466 (-$593)	-0.06	€7767 ($9988)	2	12	75	11
Sens 1: expectation maximization	-€429 (-$546)	-0.04	€10725 ($13653)	4	13	66	18
Sens 2: per-protocol	-€321 (-$409)	-0.08	€4013 ($5109)	4	25	64	8
Sens 3: healthcare perspective	€71 ($90)	-0.06	dominated^1^	8	55	33	4
Base case CUA	-€466 (-$593)	0.007	dominant^2^	8	6	26	60
Sens 1: expectation maximization	-€429 (-$546)	0.005	dominant^2^	8	8	27	56
Sens 2: per-protocol	-€323 (-$409)	-0.006	€53833 ($68532)	8	21	43	28
Sens 3: health care perspective	€71 ($90)	0.007	€10143($12913)	38	26	6	30

*Note*. Incr. Cost = Incremental costs, i.e. Cost_ACT_—CostC_BT_; Incr. Effect = Incremental effects, i.e. Effect_ACT_—EffectC_BT_; ICER = Incremental Cost Effectiveness Ratio; CEA = Cost-effectiveness analysis; CUA = Cost-utility analysis; NE = northeast quadrant with higher cost for better effects; NW = northwest quadrant with higher cost for less effect (= dominated); SW = southwest quadrant with less cost for less effect; SE = southeast quadrant with less costs for better effects (= dominant).

^1^ “Dominated”, because ACT costs more and is less effective than CBT, hence reject ACT as a cost-effective alternative for CBT

^2^ “Dominant”, because ACT costs less than CBT and has better effectiveness than CBT, hence accept ACT as the more cost-effective alternative treatment option compared to CBT.

#### Cost-effectiveness

**Fig 2:**
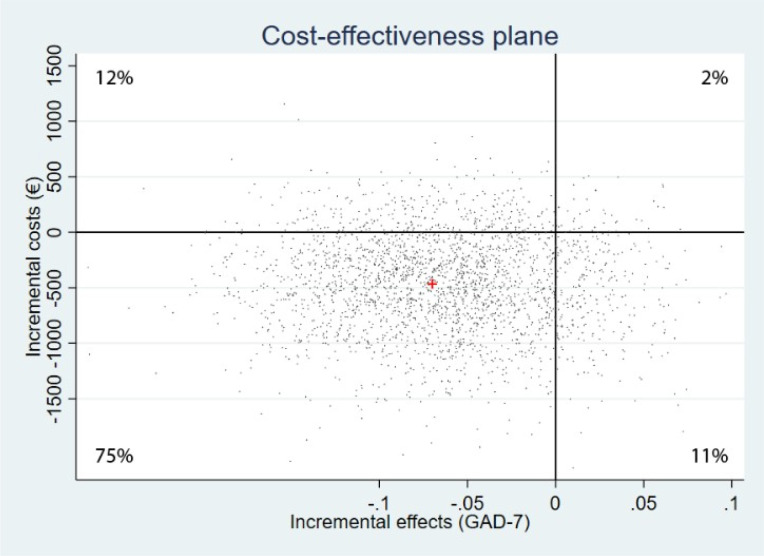
Cost-effectiveness plane reflecting the probability that blended ACT is cost-effective compared to CBT in terms of treatment responders.

In the base case cost-effectiveness analysis, the mean incremental costs and effects (treatment responders) from the 2500 bootstrapped samples were -€466 (-$593) and -0.06, which translates to an ICER of €7767. This ICER means that every treatment responder gained by offering CBT instead of blended ACT costs €7767.The incremental cost-effectiveness plane in Fig 2 shows that the large majority (75%) of the 2500 bootstrapped ICERs fell in the south-west quadrant, indicating lower costs associated with ACT compared to CBT, but also a lower treatment response rate.

The EM-imputation and per-protocol sensitivity analyses confirmed the finding from the base case analysis that compared to CBT, blended ACT generates a lower treatment response rate albeit for lower costs per treatment responder. In the cost-effectiveness planes this was reflected by a majority of 66%, respectively 64% of the bootstrapped ICERs falling into the south-west quadrant. In the analysis from the healthcare perspective, a majority of 55% of the bootstrapped ICERs fell in the northwest quadrant, indicating that from this perspective blended ACT is dominated by face-to-face CBT because it is associated with a lower treatment response rate and higher healthcare costs.

#### Cost-utility

**Fig 3:**
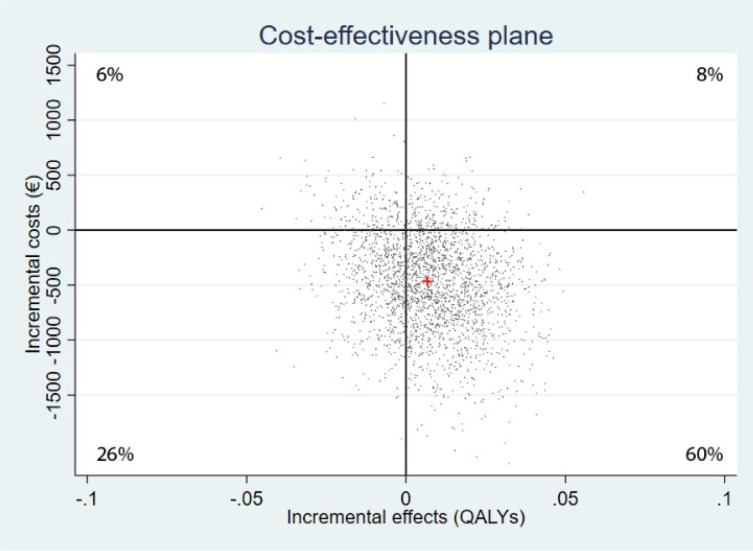
Cost-effectiveness plane reflecting the probability that blended ACT is cost-effective compared to CBT in terms of QALYs (cost-utility).

**Fig 4:**
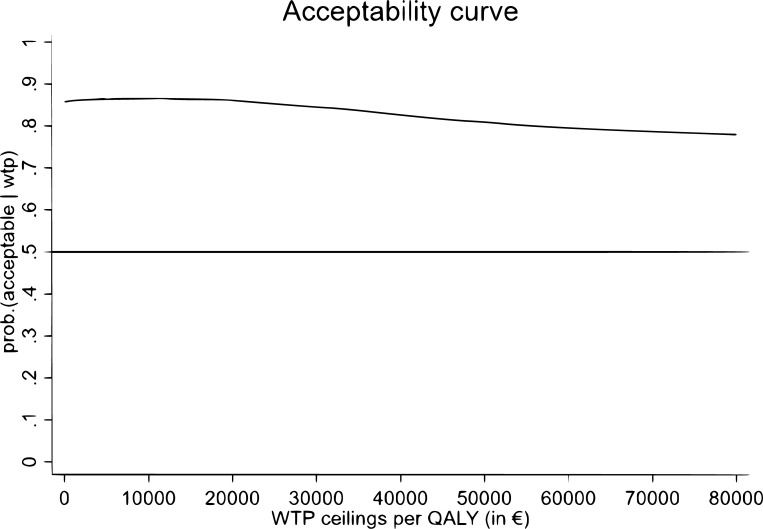
Acceptability curve reflecting the probability that blended ACT is cost-effective compared to CBT in terms of QALYs (cost-utility) at different willingness-to-pay ceilings.

In the base case cost-utility analysis ACT cost €466 ($593) less than CBT over the 12-month time period, and was associated with a QALY gain of 0.007. As can be seen in the incremental cost-effectiveness plane in Fig 3, the majority (60%) of bootstrapped ICERs fell in the south-east quadrant, indicating that in terms of cost-utility blended ACT is likely to be the dominant treatment, with lower costs and larger QALY gains compared to CBT. At the WTP ceilings of €50,000 and €80,000 per QALY the probability of ACT being cost-effective was respectively 81% and 78%, as can be seen in Fig 4.

The sensitivity analysis on the EM-imputed dataset had roughly similar results as the base case analyses, with 56% of the bootstrapped ICERs located in the south-east quadrant of the cost-effectiveness plane. The probability of ACT being cost-effective compared to CBT at WTP thresholds of €50,000 and €80,000 was 77% and 73%, respectively.

Contrary to the base case analyses, the per protocol analysis indicated less QALY gains in the ACT group than the CBT group. A fraction of 43% of the bootstrapped ICERs fell in the southwest quadrant, indicating lower costs associated with blended ACT, but also health losses. The probability of ACT being cost-effective compared to CBT at WTP ceilings of €50,000 and €80,000 was 50% and 46%, respectively.

Analysis from the healthcare perspective resulted in a total of 38% of the bootstrapped ICERs in the northeast quadrant, indicating that compared to CBT, blended ACT results in better health but for more healthcare costs. The probability of ACT being cost-effective compared to CBT was 63% at the WTP of €50,000 and 65% at the ceiling of €80,000.

## Discussion

The present study evaluated the cost-effectiveness and cost-utility of a brief blended ACT intervention compared to brief face-to-face CBT for older adults with anxiety symptoms. This health economic evaluation was conducted alongside an RCT which previously demonstrated that there were no statistically significant differences between the interventions in terms of anxiety symptom improvement at posttreatment and 12-month follow-up [22].

The results from the current study confirm the comparable effects of these interventions and do not indicate a clear preference for either the blended ACT intervention or the CBT intervention from a clinical perspective: ACT was associated with slightly fewer treatment responders on the GAD-7 and tiny QALY gains compared to CBT. The general impression therefore is that both treatments are equally effective, because the differences, if any, were statistically insignificant and clinically irrelevant. Assuming that there are virtually no clinically relevant effect differences between the interventions, blended ACT might be preferred over CBT from a strictly economic point of view. In all analyses from the societal perspective, the blended ACT intervention was associated with somewhat lower costs than CBT. In the base case analyses, 86% of the bootstrapped ICERS were indicative of lower costs and the mean per-participant societal cost reduction associated with blended ACT compared to CBT was €466 ($593). The observed costs reduction stemmed completely from the lower productivity costs in the blended ACT group and disappeared when the analyses were conducted from a healthcare perspective. When only considering healthcare costs, blended ACT was even slightly more expensive (by €71 ($90)) than CBT.

In the cost-effectiveness analyses, blended ACT was associated with slight health losses compared to CBT, but also with lower costs. The ICER of €7767 means that each treatment responder gained by offering CBT instead of blended ACT, would cost €7767. Put differently, each treatment responder lost by offering blended ACT instead of CBT would save €7767. Since there are no established willingness-to-pay thresholds for the outcome measure used in the current CEA analysis, it is not possible to tell whether this would be considered a reasonable tradeoff between health gains and costs. In terms of cost-utility, the small QALY gains combined with societal cost reductions in the ACT condition translated into a 81% and 78% probability of blended ACT being cost-effective compared to CBT at willingness-to-pay ceilings of €50,000/QALY and €80,000/QALY respectively. However, sensitivity analyses did not confirm these findings: in the per protocol analyses, the CBT group had larger QALY gains than the ACT group. It is therefore premature to conclude that blended ACT is cost-effective compared to CBT in terms of QALY’s.

The results of the current study do not allow for a decisive conclusion that from a health-economic perspective blended ACT should be preferred over CBT in the treatment of older adults with anxiety symptoms. The findings do suggest that blended ACT is associated with lower productivity costs, which is a factor that could be taken into account by healthcare providers and policy makers. For patients with an occupation (either paid or unpaid), the blended ACT intervention might be preferred over the CBT intervention as it is likely to be associated with less costs related to productivity losses. However, in practice, clinical (policy) decisions are not and should not be solely guided by economic considerations. Looking at the clinical equivalency of blended ACT and CBT for anxiety in later life, both interventions should be covered by insurance and the choice between these treatments should for now be predominantly guided by practical and medical-ethical considerations and preferences of both patient and therapist. Such a model of shared decision making, which promotes patient autonomy, can lead to improved treatment adherence and outcomes by increasing the alignment of the treatment with a patient’s preferences and values [47, 48].

The current study was the first to assess the cost-effectiveness and cost-utility of an ACT intervention compared to a CBT intervention in any patient population. Therefore, we cannot compare the current findings with previous research. Health economic evaluations of ACT and other third-wave cognitive behavioral therapies are remarkably scarce given the growing body of evidence in support of their clinical effectiveness [49]. This was also the main conclusion of a recent meta-analysis into the economic impact of third-wave cognitive behavioral therapies, which only included eleven trials, of which three were focused on ACT [49]. To bring ACT to the next stage of clinical trial testing, health economic evaluations in which ACT interventions are compared to other active treatments would be welcome.

Some limitations of the current study need to be addressed. First, a substantial number of participants dropped out of the RCT and did not complete the posttreatment and/or follow-up measurements. This resulted in a considerable amount of missing data. However, we imputed missing data using predictive mean matching and expectation maximization—two well-established imputation methods [42]-and sensitivity analyses based on both imputation techniques led to very similar results. Another limitation concerns the fact that the TIC-P only assessed participants’ healthcare use and work productivity during the four weeks preceding each measurement moment. We used linear interpolation to estimate the costs between the measurement points at months 0, 6 and 12 to obtain the cumulative costs over the full 12-month study period, but we cannot ascertain whether the assumption of linear change between the measurement points is valid. Lastly, all measures are based on self-report, which can be vulnerable to recall bias. Medication and other healthcare use is often underestimated in self-reports [28]. However, medication use was asked over a short period of two weeks retrospectively and if any bias would have occurred, then most likely in equal measure across both conditions.

Overall, the results of this health-economic evaluation in a sample of older adults with anxiety symptoms suggest that the ACT intervention and CBT intervention do not differ in terms of treatment responders and QALY gains over a one year period. The analyses indicate that, from a societal perspective, the blended ACT intervention has a small economic advantage over the CBT-intervention, because it is associated with less productivity costs. Combined with earlier findings about the comparability of the effectiveness of both interventions on multiple clinical outcomes, the current findings imply that both interventions should be covered by insurance and that -following the principles of shared decision making- clinicians and patients should collaboratively decide on which intervention they prefer, guided by personal, ethical and practical considerations.

## Supporting information

S1 Checklist CONSORT 2010 checklist of information to include when reporting a randomised trial. (DOC)

S1 Appendix Prices health care units and productivity losses. (DOCX)

S2 Appendix Indirect medical costs (travel costs). (DOCX)

S3 Appendix Total reported units of healthcare utilization and total reported days of absenteeism and presenteeism in the ACT-group and CBT-group at over assessments. (DOCX)

S4 Appendix Mean and median incremental costs and effects of the 2,500 bootstraps. (DOCX)

S5 Appendix Cost-effectiveness planes of the sensitivity analyses. (ZIP)
