# Supplementary material for: Cost-effectiveness and cost-utility of an Acceptance and Commitment Therapy intervention vs. a Cognitive Behavioral Therapy intervention for older adults with anxiety symptoms: A randomized controlled trial
Source: PLoS One. 2022 Jan 26;17(1):e0262220. doi: 10.1371/journal.pone.0262220 (PMC8791485; doi:10.1371/journal.pone.0262220)
Supplement: S1 Appendix — (DOCX) [file pone.0262220.s002.docx]

**Appendix 1. Prices health care units and productivity losses**

| Health care unit | Price* |
| --- | --- |
| Consult general practitioner | €35.24 |
| Home visit general practitioner | €53.40 |
| Telephone consult general practitioner | €18.16 |
| Consult mental health counselor at general practitioner | €18.16 |
| Consult psychotherapist/psychiatrist | €104.66 |
| Consult fysiotherapist/ergotherapist | €35.24 |
| Consult social worker | €69.42 |
| Consult company doctor | €35.24 |
| Consult medical specialist | €97.19 |
| Consult alternative medicine | €14.95 |
| Meeting selfhelp group | €14.95 |
| Visit home care service | €22.96 |
| Pharmacist dispensing costs | €6.41 |
| Daily dose medication depression | €0.13 |
| Daily dose medication anxiety/stress | €0.08 |
| Daily dose sleep medication | €0.09 |
| Daily dose pain medication | €0.67 |
| Hour paid work | €37.11 |
| Hour voluntary work / informal care | €14.95 |

*prices were calculated using standard economic prices as reported for the year 2015, indexed for the year 2019.
