# Supplementary material for: Cost-effectiveness and cost-utility of an Acceptance and Commitment Therapy intervention vs. a Cognitive Behavioral Therapy intervention for older adults with anxiety symptoms: A randomized controlled trial
Source: PLoS One. 2022 Jan 26;17(1):e0262220. doi: 10.1371/journal.pone.0262220 (PMC8791485; doi:10.1371/journal.pone.0262220)
Supplement: S2 Appendix — (DOCX) [file pone.0262220.s003.docx]

**Appendix 2. Indirect medical costs (travel costs)**

| Health care service | Price (kilometers return trip) |
| --- | --- |
| General practice / pharmacy | €0.38 (2 km) |
| Mental health care institution | €5.70 (30 km) |
| Hospital | €2.66 (14 km) |
| Fysiotherapist/ergotherapist | €0.84 (4.4 km) |
| Alternative medicine / self-help group | €3.80 (20 km) |
