# Supplementary material for: Cost-effectiveness and cost-utility of an Acceptance and Commitment Therapy intervention vs. a Cognitive Behavioral Therapy intervention for older adults with anxiety symptoms: A randomized controlled trial
Source: PLoS One. 2022 Jan 26;17(1):e0262220. doi: 10.1371/journal.pone.0262220 (PMC8791485; doi:10.1371/journal.pone.0262220)
Supplement: S3 Appendix — (DOCX) [file pone.0262220.s004.docx]

**Appendix 3.** Total reported units of healthcare utilization and total reported days of absenteeism and presenteeism in the ACT-group and CBT-group at over assessments.

|  | Baseline | | Follow-up (6 months) | | Follow-up (12 months) | |
| --- | --- | --- | --- | --- | --- | --- |
| Resource use | ACT  (n=150) | CBT  (n=164) | ACT  (n=88) | CBT  (n=112) | ACT  (n=86) | CBT  (n=96) |
| Consult GP | 73 | 59 | 36 | 45 | 28 | 27 |
| Home visit GP | 2 | 2 | 1 | 1 | 1 | 1 |
| Telephone consult GP | 19 | 18 | 5 | 12 | 2 | 5 |
| Consult GP’s mental health counselor | 19 | 19 | 11 | 21 | 11 | 14 |
| Consult psychotherapist/psychiatrist | 1 | 3 | 7 | 11 | 7 | 8 |
| Consult fysiotherapist/ergotherapist | 107 | 84 | 63 | 72 | 51 | 48 |
| Consult social worker | 4 | 0 | 1 | 2 | 1 | 0 |
| Consult company doctor | 6 | 6 | 1 | 2 | 4 | 6 |
| Consult medical specialist | 45 | 56 | 19 | 34 | 23 | 20 |
| Consult alternative medicine | 15 | 25 | 10 | 9 | 9 | 7 |
| Meeting selfhelp group | 0 | 3 | 3 | 0 | 0 | 1 |
| Visit home care service | 92 | 26 | 71 | 29 | 101 | 20 |
| Use of antidepressant | 168 | 196 | 118 | 133 | 93 | 142 |
| Use of anxiolytics | 197 | 134 | 20 | 89 | 65 | 94 |
| Used medication for sleep | 97 | 164 | 25 | 113 | 35 | 91 |
| Used medication for pain | 150 | 169 | 80 | 88 | 73 | 56 |
| Absenteeism work | 48 | 39 | 17 | 33 | 37 | 39 |
| Presenteeism work | 147 | 202 | 29 | 40 | 55 | 59 |
| Absenteeism informal care | 39 | 4 | 0 | 2 | 0 | 3 |
| Presenteeism informal care | 30 | 15 | 1 | 17 | 7 | 12 |
| Absenteeism voluntary work | 43 | 5 | 6 | 9 | 0 | 24 |
| Presenteeism voluntary work | 18 | 22 | 14 | 14 | 10 | 8 |
