# Supplementary material for: Cost-effectiveness and cost-utility of an Acceptance and Commitment Therapy intervention vs. a Cognitive Behavioral Therapy intervention for older adults with anxiety symptoms: A randomized controlled trial
Source: PLoS One. 2022 Jan 26;17(1):e0262220. doi: 10.1371/journal.pone.0262220 (PMC8791485; doi:10.1371/journal.pone.0262220)
Supplement: S4 Appendix — (DOCX) [file pone.0262220.s005.docx]

**Appendix 4. Mean and median incremental costs and effects of the 2,500 bootstraps**

| Analysis | M Incr. Cost | Mdn Incr. costs | M Incr. Effect | Mdn Incr. costs |
| --- | --- | --- | --- | --- |
| Base case CEA | -€466 | -€452 | -0.06 | -0.06 |
| Sens 1: EM imputation | -€429 | -€424 | -0.04 | -0.04 |
| Sens 2: per-protocol | -€321 | -€304 | -0.08 | -0.08 |
| Sens 3: healthcare | €71 | €61 | -0.06 | -0.07 |
| Base case CUA | -€466 | -€451 | 0.007 | 0.007 |
| Sens 1: EM Imputation | -€429 | -€424 | 0.005 | 0.005 |
| Sens 2: per-protocol | -€323 | -€304 | -0.006 | -0.006 |
| Sens 3: health care | €71 | €59 | 0.007 | 0.007 |

*Note.*Incr. Cost=Incremental costs, i.e. Cost_ACT_ - CostC_BT_; Incr. Effect=Incremental effects, i.e. Effect_ACT_ - EffectC_BT_
