# Supplementary figures and images for: Cost-effectiveness and cost-utility of an Acceptance and Commitment Therapy intervention vs. a Cognitive Behavioral Therapy intervention for older adults with anxiety symptoms: A randomized controlled trial
Source: PLoS One. 2022 Jan 26;17(1):e0262220. doi: 10.1371/journal.pone.0262220 (PMC8791485; doi:10.1371/journal.pone.0262220)

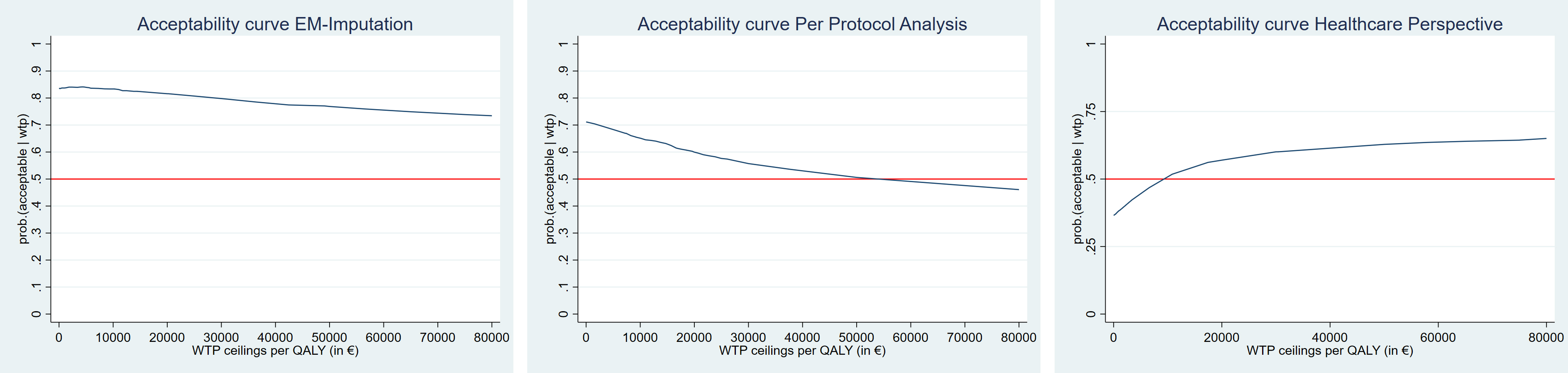

Supplement: S5 Appendix — (ZIP) [file pone.0262220.s006.zip › S5_Appendix/Appendix 5 Fig 3.jpg]
